# Association between tissue hypoxia and elevated non-protein sulphydryl concentrations in human cervical carcinoma xenografts

**DOI:** 10.1038/sj.bjc.6690797

**Published:** 1999-11

**Authors:** F Moreno-Merlo, T Nicklee, D W Hedley

**Affiliations:** Department of Oncologic Pathology, Princess Margaret Hospital, 610 University Avenue, Toronto, Ontario, M5G 2M9, Canada; Department of Medical Biophysics, University of Toronto, Ontario, Toronto, Canada

**Keywords:** hypoxia, glutathione, cervical cancer, image cytometry

## Abstract

A double staining technique was developed for the simultaneous measurement of tissue hypoxia and the concentration of non-protein sulphydryls (NPSH), based on the fluorinated nitroimidazole EF5 and the fluorescent histochemical NPSH stain 1-(4-chloromercuriphenoylazo)-naphthol-2 (mercury orange). Cryostat sections of tumour tissue were examined by fluorescence image analysis, using a computer-controlled microscope stage to generate large tiled field images of the cut tumour surface. This method was applied to the human cervical squamous cell carcinoma lines ME180 and SiHa, grown as xenografts in severe combined immunodeficient (SCID) mice, in order to determine if there is a systematic relationship between tissue hypoxia and NPSH levels. Hypoxic regions of the tumours, defined by EF5 labelling, were found to show greater NPSH concentrations relative to better oxygenated regions. This is probably due to increases in glutathione, since the ME180 and SiHa xenografts contained low levels of cysteine and metallothionein; the other major cellular thiols that can bind to mercury orange. Because the effects of glutathione on radiation and chemotherapy resistance are likely to be greater under hypoxic conditions, these results have potentially important implications for the study of resistance mechanisms in solid tumours. © 1999 Cancer Research Campaign


*British Journal of Cancer* (1999) **81**(6), 989–993
© 1999 Cancer Research Campaign
Article no. bjoc.1999.0797

### Association between tissue hypoxia and elevated non-protein sulphydryl concentrations in human cervical carcinoma xenografts

**F Moreno-Merlo[1,2], T Nicklee[1] and DW Hedley[1,2]**

[1]Department of Oncologic Pathology, Princess Margaret Hospital, 610 University Avenue, Toronto, Ontario, M5G 2M9, Canada; [2]Department of Medical Biophysics, University of Toronto, Toronto, Ontario, Canada

**Summary** A double staining technique was developed for the simultaneous measurement of tissue hypoxia and the concentration of non-protein sulphydryls (NPSH), based on the fluorinated nitroimidazole EF5 and the fluorescent histochemical NPSH stain 1-(4-chloromercuriphenoylazo)-naphthol-2 (mercury orange). Cryostat sections of tumour tissue were examined by fluorescence image analysis, using a computer-controlled microscope stage to generate large tiled field images of the cut tumour surface. This method was applied to the human cervical squamous cell carcinoma lines ME180 and SiHa, grown as xenografts in severe combined immunodeficient (SCID) mice, in order to determine if there is a systematic relationship between tissue hypoxia and NPSH levels. Hypoxic regions of the tumours, defined by EF5 labelling, were found to show greater NPSH concentrations relative to better oxygenated regions. This is probably due to increases in glutathione, since the ME180 and SiHa xenografts contained low levels of cysteine and metallothionein; the other major cellular thiols that can bind to mercury orange. Because the effects of glutathione on radiation and chemotherapy resistance are likely to be greater under hypoxic conditions, these results have potentially important implications for the study of resistance mechanisms in solid tumours. © 1999 Cancer Research Campaign

**Keywords:** hypoxia; glutathione; cervical cancer; image cytometry

Non-protein sulphydryls (NPSH), particularly glutathione, have been extensively investigated for their role in resistance to chemotherapy and radiation (Mitchell and Russo, 1987; Coleman et al, 1988; Biaglow et al, 1989; Bump and Brown, 1990; Tew, 1994). NPSH are believed to be able to interact with radiation-induced radical sites on DNA to effect their rapid repair (Bump et al, 1992; Orta et al, 1995). Although glutathione is less efficient than cysteine, it is usually present at much higher concentrations. Under aerobic conditions, DNA radical damage tends to become fixed by the binding of molecular oxygen, rather than repaired by interaction with NPSH. However, under low oxygen conditions (hypoxia), NPSH are better able to compete with oxygen for DNA radical sites, and NPSH concentrations therefore become a more important determinant of radiation sensitivity (Solen et al, 1989; Bump et al, 1992; Orta et al, 1995; Prise et al, 1998).

There is good evidence that hypoxic regions exist in some tumours, and that the presence of hypoxia is an important cause of radiation resistance in vivo. A number of clinical studies have now shown that hypoxia is associated with a poor prognosis in human solid tumours (Brizel et al, 1996, 1997; Hockel et al, 1996). A recent study from our own institution involving patients with squamous cell carcinoma of the uterine cervix found that the presence of hypoxia, as determined using the Eppendorf polarographic electrode, was associated with an inferior response to radical radiotherapy (Fyles et al, 1998).

*Received 23 February 1999*
*Revised 4 May 1999*
*Accepted 7 May 1999*

*Correspondence to:* DW Hedley

We were interested to know if there is a systematic relationship between hypoxia and NPSH levels within solid tumours, since this would be expected to modify the effects of oxygen on in vivo radiosensitivity. Recently it has become possible to identify regions of hypoxia in histological sections of solid tumours, using immunofluorescence detection of the binding of nitroimidazole compounds that undergo bioreduction to reactive intermediaries under hypoxic conditions (Lord et al, 1993). In this paper we describe a modification of this technique involving dual-labelling with the sulphydryl-reactive stain mercury orange. Stained cryostat sections obtained from human cervical cancer xenografts were examined, using a fluorescence image analysis system that is capable of mapping the whole cut surface of the tumour. Results using this technique show that NPSH levels are elevated in regions of hypoxia, suggesting that this might exacerbate the effects on radioresistance in vivo.

### MATERIALS AND METHODS

### Establishment of human cervical carcinoma xenografts

The human cervical squamous cell carcinoma cell lines ME180 and SiHa were obtained from ATCC. Xenografts were established in severe combined immunodeficient (SCID) mice by injecting the right gastrocnemius muscle with a cell suspension containing $2 \times 10^6$ cells. The tumours were allowed to grow to a size of 10–12 mm in external thigh diameter.

The fluorinated nitroimidazole compound EF5 (2-(2-nitro-1H-imidazol-l-yl)-N-(2,2,3,3,3-pentafluoropropyl)acetamide) was a gift of Dr Cameron Koch, University of Pennsylvania (Lord et al, 1993). It was made up as a 10 mM solution in 0.9% saline, and injected at a dose of 1% of body weight via a tail vein 3 h before excising the tumours. One mouse from each group of animals was used as a control and received no EF5 injection. After excision the tumours were bisected, placed in a vial containing OCT-embedding medium (Tissue-Tek, Sakura, USA), and immediately frozen and stored in liquid nitrogen.

### Double fluorescence staining with mercury orange and anti-EF5

### *NPSH staining*

Tumour tissue sections 4-μm thick were cut using a cryostat at a chamber temperature of –25°C. The method for staining non-protein sulphydryls in tissue sections was an adaptation from the single cell method previously published by our laboratory (Thomas et al, 1995), and similar to other histochemical techniques based on mercury orange (Asghar et al, 1975; Larrauri et al, 1987; Philbert et al, 1991). The sulphydryl-reactive dye 1-(4-chloromercuriphenoylazo)-naphthol-2 (mercury orange; Sigma, St Louis, MO, USA) was first dissolved in acetone, then distilled water was added to produce a final concentration of 75 μM in 9:1 (v/v) acetone-water. In order to minimize the loss of reduced thiols through oxidation, the cryostat sections were immediately stained for 5 min in the mercury orange solution in Coplin jars placed on ice. They were then rinsed twice with 9:1 acetone-distilled water, and allowed to air-dry before being fixed in 3.7% buffered formaldehyde for 10 min and then rinsed with phosphate-buffered saline (PBS) three times.

### *Anti-EF5 labelling*

The formalin-fixed, mercury orange-stained tissue sections were first blocked with 20% skimmed milk, 5% mouse serum, 1.5% albumin and 0.3% Tween. The ELK3-51 monoclonal antibody to EF5 conjugated to Cy5 was supplied by Dr Cameron Koch, University of Pennsylvania as a 1.5 mg $ml^{-1}$ solution, and used at a dilution of 1:20. The sections were incubated for 1 h and then rinsed three times with PBS. Finally, the slides were mounted using Vectorshield mounting medium (Vector Laboratories, Burlingame, CA).

### Immunohistochemical staining with EF5 monoclonal antibody

Immunohistochemical (IHC) staining for EF5 was done using the Level 2 Multispecies Ultra Streptavidin Detection System HRP kit (Signet Laboratories, Dedham, MA, USA). Serial sections to those used for the fluorescence staining method were air-dried, fixed for 10 min in 3.7% formol PBS, and rinsed three times in PBS. Endogenous peroxidases were quenched using 0.3% hydrogen peroxide for 10 min. After rinsing with PBS sections were blocked as for the immunofluorescence anti-EF5 method. The sections were then incubated for 1 h with a biotinylated anti-EF5 monoclonal antibody. After rinsing, the kit link and labelling reagents were used according to the manufacturer's instructions. Sections were then incubated with AEC chromogen for 5 min and counter-stained with Mayer's haemalum solution (BDH, Poole, UK).

### Computerized image analysis

### *Transmitted light study*

The sections stained with EF5 monoclonal antibody by IHC were studied using a MicroComputer Image Device (MCID; Imaging Research Inc., St Catharine's, Ontario, Canada) linked to a colour CCD camera (Sony DXC 970 MD) mounted on a transmitted-light microscope (Zeiss Axioskop) fitted with a Ludl Biopoint motorized stage. Using a 10× objective and an automated mini program, a microscopic field by field digitized tiled image of the entire tumour surface was obtained. These images showing the hypoxic tumour regions were used as a guide for selecting regions of the tumour for analysis by fluorescence microscopy, as described below.

### *Quantitative fluorescence microscopy*

A second MCID image analysis system was used to analyse tissue sections that were double-labelled with mercury orange and Cy5-conjugated anti-EF5. This system has similar computer hardware and software to that used for transmitted light microscopy, linked to a Xillix MicroImager (Xillix, Vancouver, Canada) mounted on a reflected fluorescence microscope (Olympus BX50) fitted with a Ludl Biopoint motorized stage.

Printouts of the immunoperoxidase staining for the entire cut surface of the tumours were used as maps to select regions encompassing approximately 20–30% of the tumour section surface for analysis. These regions were selected on the basis of consisting largely of viable tumour tissue containing clearly delineated hypoxic microregions. Using a 20×, 0.5 N.A. objective lens, tiled field images of these region were obtained, based on 40–80 individual fields. The slide was first scanned using red illumination, to show the areas of hypoxia as detected by the Cy5 conjugated anti-EF5 antibody. Next, a tiled field image of the same regions was obtained using an excitation filter centred at 540 nm which optimally excites mercury orange. Fluorescence emission was collected using a 585/40 nm bandpass filter. To study the relationship between hypoxia and levels of NPSH, the EF5 and mercury orange images were visualized in two separate but linked digitized channels on the system monitor. Rectangles measuring 300 × 100 μm were drawn on the EF5 image in areas that consisted of viable tumour tissue that were either positively labelled with anti-EF5, or uniformly EF5-negative. To eliminate bias these regions were selected without reference to the mercury orange image. Because mercury orange fluorescence was much higher in tumour tissue compared to stroma, it was possible to set a threshold level that masked only tumour cells. The corresponding relative fluorescence intensity was then obtained. This was expressed as mean integrated optical density (IOD), on a greyscale of zero (black) to 1024 (white). Background due to autofluorescence and instrument noise was obtained by examining unstained slides fixed in 90% acetone, and subtracted from the mercury orange IOD values. For each tumour a total of ten hypoxic and ten oxygenated regions was examined.

### RESULTS

### Characterization of ME180 and SiHa xenografts

Histological examination of tissue sections stained with haematoxylin and eosin showed keratinizing, well-differentiated cervical squamous cell carcinoma in the ME180 xenografts, with very little necrosis. The SiHa xenografts also showed typical features of squamous cell carcinoma, but compared to the ME180 tumours these were poorly differentiated tumours containing areas of necrosis. Immunohistochemical staining for metallothionein, which is usually the most abundant protein sulphydryl in

 © 1999 Cancer Research Campaign

**Table 1** Me 180 tumours

| | MO-IOD in EF5-positive regions[a] | | MO-IOD in EF5-negative regions[a] | | |
|---|---|---|---|---|---|
| | **Mean** | **s.d.** | **Mean** | **s.d.** | ***P*-value[b]** |
| 1 | 361 | ±46 | 168 | ±72 | < 0.0001 |
| 2 | 394 | ±89 | 275 | ±57 | < 0.0001 |
| 3 | 314 | ±72 | 221 | ±59 | 0.004 |
| 4 | 222 | ±43 | 128 | ±47 | < 0.0001 |

[a]Mercury orange staining in ten EF5-positive and ten EF5-negative regions of each tumour. [b]Student's *t*-test.

**Table 2** SiHa tumours

| | MO-IOD in EF5-positive regions[a] | | MO-IOD in EF5-negative regions[a] | | |
|---|---|---|---|---|---|
| | **Mean** | **s.d.** | **Mean** | **s.d.** | ***P*-value[b]** |
| 1 | 160 | ±29 | 124 | ±14 | 0.002 |
| 2 | 210 | ±16 | 185 | ±18 | 0.006 |
| 3 | 165 | ±37 | 164 | ±44 | 0.917 |
| 4 | 172 | ±18 | 141 | ±9 | < 0.001 |

[a]Mercury orange staining in ten EF5-positive and ten EF5-negative regions of each tumour. [b]Student's *t*-test.

mammalian cells, showed low to undetectable levels of expression in the ME180 and SiHa xenografts.

### Mapping hypoxic microregions by EF5 IHC

Composite images showing representative examples of ME180 and SiHa xenografts are shown in Figure 1. Areas of viable tumour tissue that were positively labelled with anti-EF5 could be clearly distinguished from EF5-negative areas in all of the ME180 and SiHa xenografts examined. Surveying the entire tumour surface revealed a complex, heterogeneous distribution of hypoxia. Regions of viable tumour tissue could be identified up to 1 mm across in which there was little or no EF5-labelling. Other regions that were morphologically similar in parallel haematoxylin and eosin sections showed highly evolved patterns of EF5 positivity, with scalloped bands of hypoxic tissue interspersed with regions of EF5-negative tumour tissue. In the example of an ME180 tumour shown in Figure 1, these bands of hypoxic tissue can be seen to form ovoid structures with diameters of 300–500 µM. These EF5-positive structures were also apparent in the SiHa tumours, although less clearly delineated.

### Identification of hypoxic microregions by EF5 immunofluorescence

A much slower rate of data acquisition imposed a practical limitation on the extent of tumour tissue that could be examined by the fluorescence microscopy technique. Based on the results of the parallel sections stained for EF5 using IHC, regions of viable tumour tissue were chosen for analysis that included significant amounts of EF5-positive tissue. As illustrated in Figure 2A, the pattern of EF5-labelling detected by immunofluorescence corresponded closely to that seen using IHC, with finer detail in terms of textural features and dynamic range.

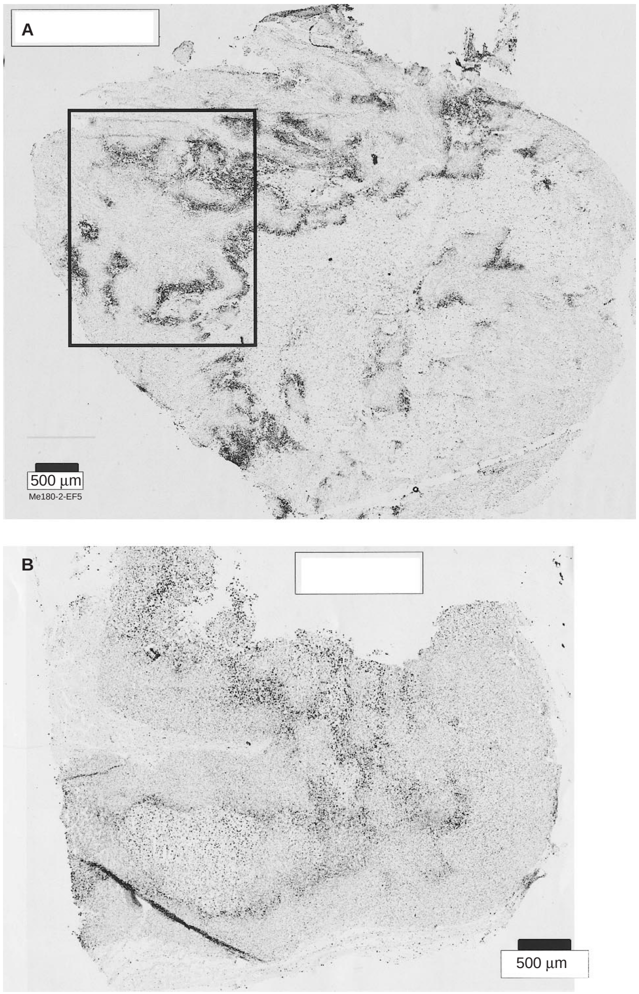

**Figure 1** Anatomical distribution of hypoxia in ME180 (**A**) and SiHa (**B**) xenografts, measured using immunohistochemical labelling of EF5. Each image is a composite built up from approximately 100 microscope fields obtained using a 10× objective lens

### Measurement of NPSH using mercury orange

Rinsing the cryostat sections in 500 µM mercuric chloride for 30 s prior to staining in mercury orange, in order to block free sulphydryl groups, decreased the fluorescence intensity to that of the autofluorescence background. This indicates that under the staining conditions used, mercury orange was specifically labelling free thiols. Time course experiments using cryostat sections showed that the mercury orange staining intensity increased rapidly over the first 5 min, consistent with previous work showing a high affinity for reactive sulphydryls, followed by a much slower increase in mercury orange staining which was probably due to binding to less chemically reactive protein thiols (Asghar et al, 1975; Thomas et al, 1995). There was no detectable loss of mercury orange staining during the immunofluorescence staining procedure using anti-EF5. Examination of tissue sections stained only with Cy5 conjugated anti-EF5 showed that the 585/40 nm bandpass filter used to collect mercury orange fluorescence excluded spillover from Cy5 fluourescence.

Histochemical staining of cryostat sections with mercury orange was greater in viable tumour tissue compared to necrotic tissue or stromal tissue. As can be seen in Figure 2B, mercury orange

© 1999 Cancer Research Campaign

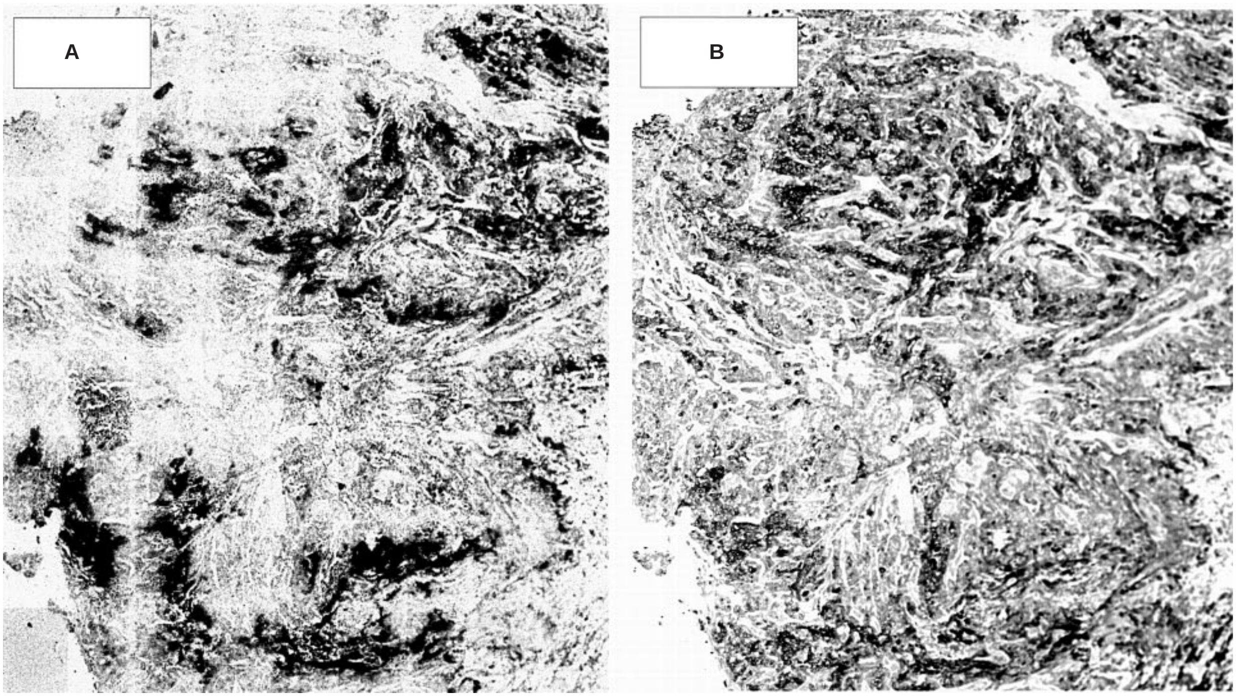

**Figure 2** Double fluorescence labelling for EF5 (**A**) and NPSH (**B**). These monochromatic images are composites of 64 individual microscope fields acquired using a 20× objective lens, taken from a cryostat section cut parallel to the immunoperoxidase-stained example shown in Figure 1. The area corresponds to the rectangle marked on Figure 1. For clarity the images have been inverted and contrast-enhanced. The rectangular regions drawn on these images represent an area measuring 300 × 100 µm.

staining tended to be stronger in regions of hypoxia defined by EF5 binding, although the concordance between the two images was not exact, with relatively high NPSH values being seen in some regions of viable tumour tissue that were EF5 negative. The correlations between hypoxia and NPSH levels were quantified by measuring the mean optical densities of mercury orange staining in EF5-positive and EF5-negative regions of four ME180 and four SiHa tumours. As shown in Tables 1 and 2, hypoxic microregions of ME180 showed an approximately 50% increase in NPSH levels compared to EF5-negative regions. This was shown to be highly significant using Student's *t*-test to compare values obtained from ten EF5-positive and ten EF5-negative regions, randomly selected from each tumour. A similar trend was observed in the poorly differentiated SiHa tumours, although this was less pronounced than in ME180 tumours.

### DISCUSSION

Because NPSH can compete with oxygen for radical sites on DNA, the question of whether there is a systematic relationship between NPSH levels and hypoxia within solid tumours is of considerable radiobiological interest. In this paper we describe a technique for simultaneous in situ measurements of tissue hypoxia and NPSH levels that can address this question directly. Using this technique we found significantly greater NPSH levels in hypoxic microregions relative to better oxygenated regions of xenografts established from human cervical squamous cell carcinoma lines.

The sulphydryl stain mercury orange reacts more rapidly with active thiols such as glutathione and cysteine than with protein sulphydryls, so that under controlled labelling conditions it is fairly specific for NPSH. We have previously shown that the staining method used in these experiments gives a fluorescence intensity that is closely correlated with the biochemically-determined cellular glutathione content (Thomas et al, 1995). Using high-performance liquid chromatography we have found that the cysteine concentrations of ME180 and SiHa xenografts is < 10% of that of glutathione (unpublished data). Because of its exposed mercury atom, it is likely that mercury orange can also bind to metallothionein; a cysteine-rich small protein that is expressed at high levels in some cells. However, by IHC metallothionein levels were low to undetectable in the ME180 and SiHa xenografts. Blockade of free thiols with mercuric chloride resulted in almost complete loss of mercury orange staining, indicating that binding to other chemical groups is minimal under the conditions used. Therefore it is likely that the mercury orange staining of the tumours is mainly due to glutathione.

Because the reaction product between NPSH and mercury orange is retained during immunofluorescence staining, we were able to combine NPSH measurements with the detection of hypoxia using EF5. This fluorinated nitroimidazole undergoes a series of single electron reductions to reactive intermediaries that can bind to cellular macromolecules. In the presence of oxygen these reduction products are back-oxidized to EF5, and no tissue binding takes place. The partial pressure of oxygen at which bound metabolites are reduced by half corresponds to oxygen concentrations that are radiobiologically significant (i.e. approximately 5 mmHg) (Lord et al, 1993). Studies using oxygen sensitive polarographic electrodes have shown that the distribution of hypoxia within solid tumours is very heterogeneous. The introduction of IHC techniques based on compounds such as EF5 allows this heterogeneity to be visualized in the context of tissue architecture. By using a computer-controlled microscope stage and an image analysis program to fuse together multiple individual fields, we have been able to survey the distribution of hypoxia throughout

 *© 1999 Cancer Research Campaign*

the cut surface of the tumour. As seen in Figure 1, this shows a complex, heterogenous picture, with regions of the tumour appearing to be uniformly well-oxygenated, interspersed with regions showing highly evolved patterns of EF5 binding. An underlying motif is the existence of arcs of anti-EF5 staining having a radius of approximately 150–250 μM. These probably represent vascular cords; cylinders of well oxygenated tissue around a blood vessel, surrounded by a rim of hypoxia beyond the oxygen diffusion distance in tissue. This pattern is particularly seen in the well-differentiated ME180 tumours.

Hypoxic regions of ME180 tumours showed significantly higher NPSH values than oxygenated regions. A similar effect was seen in three of the four SiHa tumours examined, although technically these were more difficult to study because of the presence of necrotic tissue interspersed among viable tumour tissue, and because the EF5-positive regions tended to be less clearly delineated than in the ME180 tumours. The most likely explanation for these findings is that glutathione levels are increased in hypoxic regions of the tumours, since the other potential candidate molecules cysteine and metallothionein are present at low levels in these two tumours.

Increases in cellular glutathione content have been observed during tumour cell growth in multicellular spheroids (Romero et al, 1997), and following exposure of cells to hypoxia followed by reoxygenation in vitro (O'Dwyer et al, 1994). In this paper we now provide evidence that glutathione levels can also be increased in association with hypoxia in vivo. It is not clear from our experiments, or from the previously published data, if these increases in glutathione are the direct result of hypoxia, or occur in response to oxidative stress that can occur as a result of acute hypoxia followed by reoxygenation. The effects of glutathione and other NPSH on in vitro radiation sensitivity are relatively small under aerobic conditions, but become more significant under hypoxia (Bump et al, 1992). The increases in NPSH associated with hypoxia in cervical cancer xenografts might therefore be sufficient to produce clinically relevant increases in radiation resistance in vivo.

We next plan to study biopsy samples obtained from cervix cancer patients treated in an upcoming clinical trial of EF5, in order to establish if increased NPSH values are also seen in hypoxic regions of primary human cancers. Future laboratory studies will investigate the underlying mechanisms of the increase in NSPH seen in hypoxic regions of cervical cancer xenografts, and test if thiol-modulating agents such as buthionine sulphoximine show differential effects on hypoxic versus oxic micro-regions of solid tumours.

### ACKNOWLEDGEMENTS

We wish to thank Dr Cameron Koch, Department of Radiation Oncology, University of Pennsylvania, for supplying us with EF5 and the Cy5 and biotinylated anti-EF5 antibodies, and for many helpful discussions about these experiments. This work was supported by the National Cancer Institute of Canada with funds raised by the Terry Fox Run.

### REFERENCES

Asghar K, Reddy BG and Krishna G (1975) Histochemical localization of glutathione in tissues. *J Histochem Cytometry* **23**: 774–779

Biaglow JE, Varnes ME, Epp ER, Clark EP, Tuttle SW and Held KD (1989) Role of glutathione in the aerobic radiation response. *Int J Radiation Oncol Biol Phys* **16**: 1311–1314

Brizel DM, Scully SP, Harrelson JM, et al (1996) Tumor oxygenation predicts for the likelihood of distant metastases in human soft tissue sarcoma. *Cancer Res* **56**: 941–943

Brizel DM, Sibley GS, Prosnitz LR, Scher RL and Dewhirst MW (1997) Tumor hypoxia adversely affects the prognosis of carcinoma of the head and neck. *Int J Radiat Oncol Biol Physics* **38**: 285–289

Bump EA and Brown JM (1990) Role of glutathione in the radiation response of mammalian cells in vitro and in vivo. *Pharmac Ther* **47**: 117–136

Bump EA, Cerce BA, Al-Sarraf R, Pierce SM and Koch CJ (1992) Radioprotection of DNA in isolated nuclei by naturally occurring thiols at intermediate oxygen tension. *Radiat Res* **132**: 94–104

Coleman CN, Bump EA and Kramer RA (1988) Chemical modifiers of cancer treatment. *J Clin Oncol* **6**: 709–733

Fyles AW, Milosevic M, Wong R, et al (1998) Oxygenation predicts radiation response and survival in patients with cervix cancer. *Radiother Oncol* **48**: 149–156

Hockel M, Schlenger K, Aral B, Mitze M, Schaffer U and Vaupel P (1996) Association between tumor hypoxia and malignant progression in advanced cancer of the uterine cervix. *Cancer Res* **56**: 4509–4515

Koch CJ and Evans SM (1996) Cysteine concentrations in rodent tumors: unexpectedly high values may cause therapy resistance. *Int J Cancer* **67**: 661–667

Larrauri A, Lopez P, Lechon-Gomez MJ and Castell JV (1987) A cytochemical stain for glutathione in rat hepatocytes cultured on plastic. *J Histochem Cytochem* **35**: 271–274

Lord EM, Harwell L and Koch CJ (1993) Detection of hypoxic cells by monoclonal antibody recognizing 2-nitroimidazole adducts. *Cancer Res* **53**: 5721–5726

Meister A (1991) Glutathione deficiency produced by inhibition of its synthesis, and its reversal: applications in research and therapy. *Pharmac Ther* **51**: 155–194.

Mitchell JB and Russo A (1987) The role of glutathione in radiation and drug induced cytotoxicity. *Br J Cancer* **55**: 96–104

O'Dwyer PJ, Yao K-S, Ford P, Godwin AK and Clayton M (1994) Effects of hypoxia on detoxicating enzyme activity and expression in HT29 colon adenocarcinoma cells. *Cancer Res* **54**: 3082–3087

Orta T, Eady JJ, Peacock JH and Stell GG (1995) Glutathione manipulation and the radiosensitivity of human tumour and fibroblast cell lines. *Int J Radiat Biol* **68**: 413–419

Philbert MA, Beiswanger CM, Waters DK, Reuhl KR and Lowndes HE (1991) Cellular and regional distribution of reduced glutathione in the nervous system of the rat: histochemical localization by mercury orange and o-phthaldialdehyde-induced histofluorescence. *Toxicol Appl Pharmacol* **107**: 215–227

Prise KM, Gillies NE and Michael BD (1998) Evidence for a hypoxic fixation reaction leading to the induction of ssb and dsb in irradiated DNA. *Int J Radiat Biol* **74**: 53–59

Romero FJ, Zukowski D and Mueller-Klieser W (1997) Glutathione content of V79 cells in two- or three-dimensional culture. *Am J Physiol* **272**: C1507–C1512

Slater AFG, Stefan C, Nobel I, van den Dobbelsteen DJ and Orrenius S (1996) Intracellular redox changes during apoptosis. *Cell Death Differ* **3**: 57–62

Solen G, Edgren M, Scott OC and Revesz L (1989) Cellular glutathione content and K values. *Int J Radiat Biol* **55**: 201–210

Tew KD (1994) Glutathione-associated enzymes in anticancer drug resistance. *Cancer Res* **54**: 4313–4320

Thomas M, Nicklee T and Hedley DW (1995) Differential effects of depleting agents on cytoplasmic and nuclear non-protein sulphydryls: a fluorescence image cytometry study. *Br J Cancer* **72**: 45–50

© 1999 Cancer Research Campaign 
